# Supplementary material for: β-Lactam Pharmacokinetic/Pharmacodynamic Target Attainment in Intensive Care Unit Patients: A Prospective, Observational, Cohort Study
Source: Antibiotics (Basel). 2023 Aug 5;12(8):1289. doi: 10.3390/antibiotics12081289 (PMC10451857; doi:10.3390/antibiotics12081289)

Title: *ß*-Lactam Pharmacokinetic/Pharmacodynamic Target Attainment in Intensive Care Unit Patients: A Prospective Observational Cohort Study

Table S1. Repartition of isolated pathogens according to antimicrobial agent and corresponding ECOFFs (Eucast Epidemiological Cut off).

Isolated pathogens	Cefepime, n [ECOFF, mg/L]	Cefotaxime, n [ECOFF, mg/L]	Ceftazidime, n [ECOFF, mg/L]	Meropenem, n [ECOFF, mg/L]	Total*, n (%)
Citrobacter koseri	2 [0.125]	1 [NA]	-	-	3 (2.0)
Enterobacter aerogenes	7 [NA]	-	-	1 [NA]	8 (5.2)
Enterobacter cloacae	9 [0.125]	1 [0.5]	-	1 [0.125]	11 (7.2)
Enterococcus faecalis	2 [ND]	-	-	-	2 (1.3)
Enterococcus faecium	1 [ND]	2 [NA]	1 [NA]	2 [8]	6 (3.9)
Escherichia coli	6 [0.125]	23 [0.25]	3 [0.5]	8 [0.125]	40 (26.1)
Haemophilus influenzae	1 [0.25]	-	1 [0.5]	-	2 (1.3)
Klebsiella pneumoniae	3 [0.125]	12 [0.25]	6 [0.5]	-	21 (13.7)
Klebsiella oxytoca	1 [0.125]	-	-	-	1 (0.65)
Morganella morganii	-	1 [ND]	-	1 [0.25]	2 (1.3)
Pseudomonas aeruginosa	5 [8]	3 [32]	16 [8]	2 [2]	26 (17.0)
Proteus mirabilis	1 [0.125]	2 [0.064]	-	-	3 (2.0)
Serratia marcescens	3 [0.125]	-	-	1 [NA]	4 (2.6)
Staphylococcus aureus	1 [8]	7 [4]	1 [NA]	1 [NA]	10 (6.5)
Streptococcus pneumoniae	-	5 [0.064]	-	-	5 (3.3)
Achromobacter xylodans	-	-	1 [NA]	-	1 (0.65)
Acinetobacter pitii	-	-	1 [NA]	-	1 (0.65)

Raoultella ornithicolithic	-	-	-	1 [NA]	1 (0.65)
Streptococcus constellatus	-	1 [NA]	1 [NA]	-	2 (1.3)
Stenotrophomonas maltophila	2 [ND]	-	2 [ND]	-	4 (2.6)
ND : not determined, NA : not available					

*Some patients presented co-infection

Table S2. Target attainment at D1, D4 and D7, for documented infections (n=136).

		**Patient target attainement (%)**					
		**Day 1**	**n**	**Day 4**	**n**	**Day 7**	n
**Cefepime**	underdosed	39		31		23	
	normodosed	52	31	55	29	54	22
	overdosed	9		14		23	
**Cefotaxime**	underdosed	4		18		15	
	normodosed	89	47	82	40	85	26
	overdosed	7		-		-	
**Ceftazidime**	underdosed	48		50		26	
	normodosed	32	25	50	20	37	26
	overdosed	20		-		37	
**Meropenem**	underdosed	16		12		7	
	normodosed	47	19	53	17	65	14
	overdosed	37		35		28	

Table S3. Covariates associated with target attainment in patient presented dosage adjustment or not (univariate analysis).

	Patient target attainment	All patients at D1 n= 173	*p*	Patients at D4* without dosage adjustment between D1 and D4, n=92	n	*p*	Patients at D4* with dosage adjustment between D1 and D4, n= 41	n	*p*	Patients at D7** without dosage adjustment between D4 and D7, n= 71	n	*p*	Patients at D7** with dosage adjustment between D4 and D7, n=29	n	*p*
Weight	underdosed	73.0 (±15.1)		82,2 (±16,5)	19		74,4 (±12,3)	5		81,2 (±18,7)	17		74,0 (±6,7)	4	
	normodosed	75.8 (±12.9)	*0.084*	75,5 (±15,6)	17	*0,261*	70,6 (±13,3)	11	*0.571*	76,7 (±13,4)	13	*0.657*	110 (±74,8)	5	*0.464*
	overdosed	83.4 (±10.9)		-	1		-	0		-	0		-	1	
Hematochrite	underdosed	29.5 (±5.9)		29,3 (±4,2)	45		31,4 (±6,6)	16		30,4 (±6,4)	33		28,3 (±4,6)	12	
	normodosed	28.9 (±5.4)	*0.399*	28,0 (±5,7)	41	*0,175*	29,1 (±4,1)	20	*0.457*	28,9 (±3,7)	30	*0.760*	26,8 (2,6)	14	*0.165*
	overdosed	27.2 (±3.4)		28,6 (±4,5)	5		27,5 (6,5)	4		28,2 (±2,9)	6		31,5 (±2,1)	2	
Albuminemia	underdosed	28.7 (±4.9)		29,7 (±5,0)	43		30,5 (±4,1)	15		31,0 (±5,7)	30		31,8 (±4,3)	10	
	normodosed	28.2 (±5.4)	*0.342*	27,3 (±5,0)	35	*0,114*	26,6 (±5,1)	16	***0.032***	28,3 (±3,5)	26	*0.096*	26,5 (±4,5)	11	*0.05*
	overdosed	26.6 (±5.1)		28,3 (±1,2)	5		26,2 (±3,4)	3		27,0 (±7,1)	5		29,9 (±0,8)	2	
Creatinine	underdosed	59.2 (±34.8)		59,6 (±39,2)	45		48,9 (±14,9)	16		69,1 (±95,2)	33		41,8 (±18,5)	11	
	normodosed	86.8 (±52.1)	***<0.0001***	82,2 (±77,0)	42	***0.001***	91,2 (±80,5)	19	***0.009***	84,5 (69,4)	30	***0.0009***	80,5 (±70,0)	14	*0.144*
	overdosed	174 (±115)		170 (±65,3)	5		129 (±56,3)	4		156 (±81,5)	6		80,5 (±54,4)	2	
Indexed creatinine clearance	underdosed	106.0 (±52.2)		90,3 (±41,5)	33		97,3 (±39,0)	13		87,7 (±50,0)	23		78,5 (±31,1)	7	
	normodosed	69.8 (±37.2)	***<0.0001***	75,9 (±51,9)	31	***0.010***	67,6 (±46,2)	14	*0.071*	68,5 (±44,8)	25	***0.01***	69,8 (±55,4)	9	*0,77*
	overdosed	28.5 (±22.9)		21,6 (± 6,9)	3		28,9 (±16,3)	2		15,4 (±15,5)	4		43,2 (±41,0)	2	

Data are presented as mean ±SD

* Patients with concentration data at D1 and D4 (n=133)

** Patients with concentration data at D4 and D7 (n=100)

Table S4. Covariates associated with target attainment (multivariate analysis).

	Parameter Estimate [95% CI]	Standard error	p-value
Albuminemia	0.023 [-0.082 ; 0.129]	0.054	0.6648
Indexed creatinine clearance	0.039 [0.017 ; 0.062]	0.012	0.0007
Créatinine	-0.003 [-0.009 ; 0.004]	0.003	0.4016

Table S5. Protocol of ß-Lactam administration**.**

β-Lactams	Loading dose (gr per 30 min)	Maintenance dose (gr per day)			
		Normal renal function	Moderate renal impairment (CL_{CR} 30-60 mL/min)	Severe renal impairment (CL_{CR} <30 mL/min)	Renal replacement therapy (RRT)
**Cefepime**	2	4	1	0.5	4
**Cefotaxime**	2	6	6	1	4
**Ceftazidime**	2	6	1	0.5	2 (4 for high dose, RRT≥25mL/kg/h)

**Meropenem**	2	4	2	1	2
CL_{CR} : creatinine clearance					

Table S6: MIC threshold and ß-Lactam defined target concentrations.

Antibiotic	Recommended target concentration	MIC threshold
**Cefotaxime**	Css= 20-60 mg/L	4 mg/L (ECOFF *S. aureus*)
**Cefepime**	Css= 40-60 mg/L*	8 mg/L (ECOFF *P. aeruginosa*)
**Ceftazidime**	Css= 40-80 mg/L	8 mg/L (ECOFF *P. aeruginosa*)
**Meropenem**	Css= 10-20 mg/L	2 mg/L (ECOFF *P. aeruginosa*)

Css: target total steady state concentration following continuous administration
MIC: minimum inhibitory concentration
ECOFF: epidemiological cut-off (EUCAST. Available online: URL (accessed on 03/10/2018). https://www.eucast.org/mic_and_zone_distributions_and_ecoffs, 12/2020)
* A narrower therapeutic index was considered for cefepime due to its important neurotoxicity ability [23,28-29]

Figure S1. Evolution over time of target attainment (%) in the different groups: no dosage adjustment, adjustment D1-D4, adjustment D1-D4 and D4-D7, and adjustment D4-D7 (patients with concentration data at D1, D4 and D7; n=95).

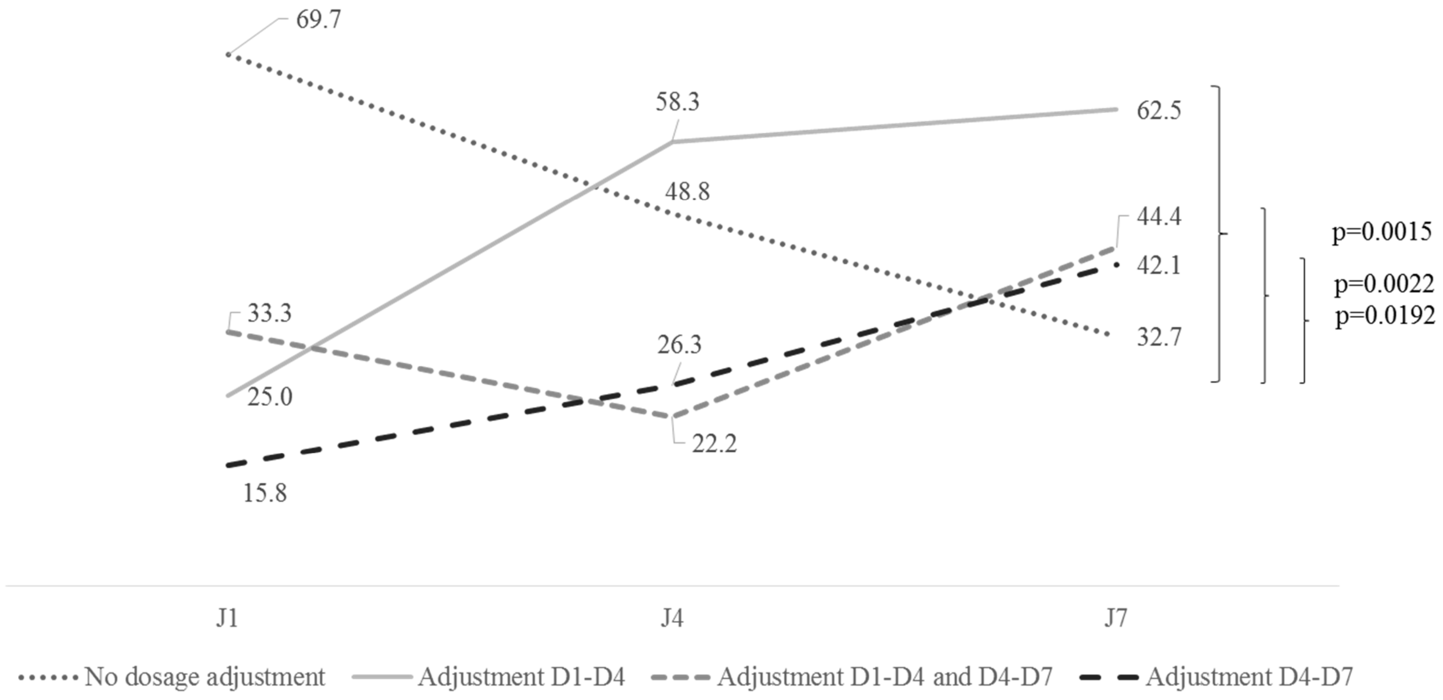

Supplement: Supplementary file 1 [file antibiotics-12-01289-s001.zip › antibiotics-2529104-supplementary.pdf]
